# Fluorine-18 Fluorocholine Positron Emission Tomography/Computed Tomography in Primary Hyperparathyroidism: A Case Report and Review of Literature

**DOI:** 10.7759/cureus.21958

**Published:** 2022-02-06

**Authors:** Diogo Ramalho, Gustavo Rocha, José M Oliveira, Maria J Oliveira

**Affiliations:** 1 Endocrinology, Centro Hospitalar de Vila Nova de Gaia/Espinho, Vila Nova de Gaia, PRT; 2 Nuclear Medicine, Atrys Health, Santa Maria da Feira, PRT

**Keywords:** technetium tc 99m sestamibi, fluorine-18 fluorocholine, parathyroidectomy, parathyroid neoplasms, primary hyperparathyroidism

## Abstract

Positron emission tomography (PET) tracers (Fluorine-18 Fluorocholine [^18^F-Fluorocholine] and Carbon-11 Choline [^11^C-Choline]) have been widely used with promising accuracy in detecting abnormal parathyroids, being crucial for an effective and safe minimally invasive parathyroidectomy. We report a case of a 72-year-old woman with a long-term personal history of osteoporosis and recurrent nephrolithiasis with the need for invasive interventions. Primary hyperparathyroidism was biochemically assumed, although localization of the hyperfunctioning parathyroid had been challenging since cervical ultrasound and technetium-99m sestamibi scintigraphy were negative/equivocal. An ^18^F-Fluorocholine positron emission tomography/computed tomography (PET/CT) was performed, having identified a small cervical nodule with increased tracer uptake, compatible with a right parathyroid adenoma. After its removal, the patient went into clinical and biochemical remission. ^18^F-Fluorocholine PET/CT allowed an effective and safe parathyroidectomy as conventional imaging modalities were inaccurate in detecting the abnormal parathyroid, in this patient with serious hyperparathyroidism-related complications.

## Introduction

Primary hyperparathyroidism (HPT) is a common endocrinopathy, which develops as a consequence of autonomous production of parathyroid hormone (PTH), most frequently by a solitary parathyroid adenoma (around 90%) [1]. It is usually treated with parathyroidectomy, which with technical development has become minimally invasive [2]. A minimally invasive parathyroidectomy requires effective preoperative localization of the abnormal parathyroid gland. Therefore, in order to improve localization accuracy of parathyroid adenomas and surgery efficacy, positron emission tomography (PET) tracers (Fluorine-18 Fluorocholine [^18^F-Fluorocholine], Carbon-11 Choline [^11^C-Choline]) have been increasingly used with promising outcomes, apart from the conventional Technetium-99m sestamibi (^99m^Tc-sestamibi) scintigraphy [3].

## Case presentation

We describe a case of a 72-year-old woman with a 10-year personal history of osteoporosis of the lumbar spine and recurrent nephrolithiasis in a current single functioning kidney (left radical nephrectomy due to a renal cell carcinoma in 2016) and stage 2 chronic kidney disease (serum creatinine 0.81 mg/dL; estimated glomerular filtration rate [eGFR] 71.6 mL/min/1.73 m²). A total of three sessions of extracorporeal shock wave lithotripsy (ESWL) were performed until the nephrectomy. After the surgery, right nephrolithiasis recurred and the patient needed a double J ureteral catheterization and one session of ESWL. The aforementioned clinical picture led to the investigation of a possible phospho-calcic metabolism disorder. A presumptive biochemical diagnosis of a primary HPT was made at that time, although associated with a severe vitamin D deficiency as it is displayed in Table 1. The patient was euthyroid.

**Table 1:** Initial laboratory findings

Parameters	Patient´s value	Reference range
Albumin-corrected serum calcium (mg/dL)	10.6	8.8-10.2
Phosphate (mg/dL)	1.7	2.7-4.5
Magnesium (mEq/L)	1.6	1.3-2.1
Parathyroid hormone (pg/mL)	154.0	15.0-65.0
25-hydroxyvitamin D (mmol/L)	25.0	62.5-200.0
Creatinine (mg/dL)	0.84	0.51-0.95
Thyroid-stimulating hormone (μIU/mL)	1.59	0.27-4.20
Free T4 (ng/mL)	1.16	0.93-1.70
24-h urinary calcium (mg/24h)	413	100-320

At three-month follow-up, despite correction of the vitamin D deficiency using oral cholecalciferol, serum PTH remained elevated (Table 2), which reinforced the diagnosis of a primary HPT with surgical criteria (osteoporosis of the lumbar spine; nephrolithiasis) [4].

**Table 2:** Three-month follow-up laboratory results

Parameters	Patient´s value	Reference range
Albumin-corrected serum calcium (mg/dL)	10.5	8.8-10.2
Phosphate (mg/dL)	1.9	2.7-4.5
Parathyroid hormone (pg/mL)	165.0	15.0-65.0
25-hydroxyvitamin D (mmol/L)	75.0	62.5-200.0

**Figure 1:**
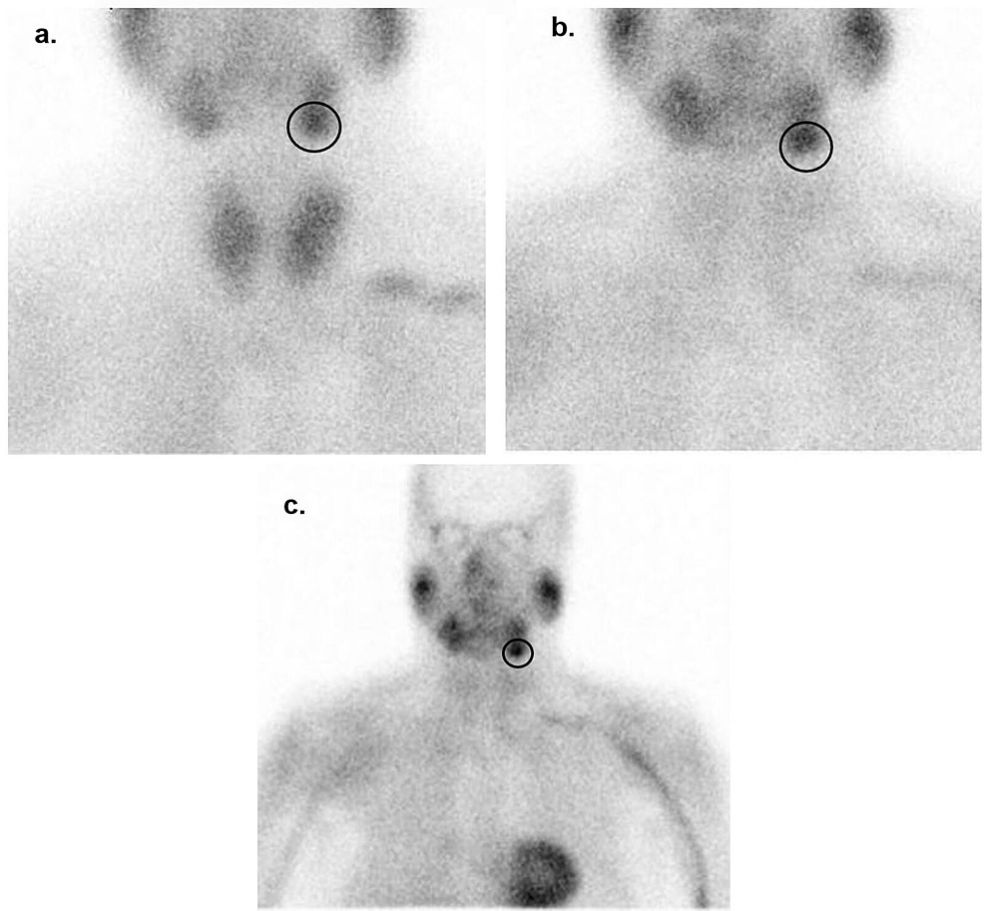
Technetium-99m sestamibi scintigraphy After administration of the radiopharmaceutical, (a) 10 minutes; (b) and (c) three hours. Black circles – pronounced uptake on the inferior region of the left submandibular gland.

Localization of the hyperfunctioning parathyroid tissue was then initiated. Neck ultrasound demonstrated a multinodular goiter, with multiple isoechoic solid nodules being identified in both lobes, the largest one in the left lobe with 23 mm of longitudinal diameter. The other were pericentimetric nodules with no suspicious features of malignancy. No submandibular or laterocervical adenopathies and no suspected parathyroid lesions were found. The patient performed fine-needle aspiration cytology of the left thyroid nodule, which was “benign” (Bethesda II). Several ^99m^Tc-sestamibi scintigraphies evidenced a more pronounced uptake on the left submandibular gland (Figures 1a-1c), with no significant tracer uptake in the typical parotid gland localization.

**Figure 2:**
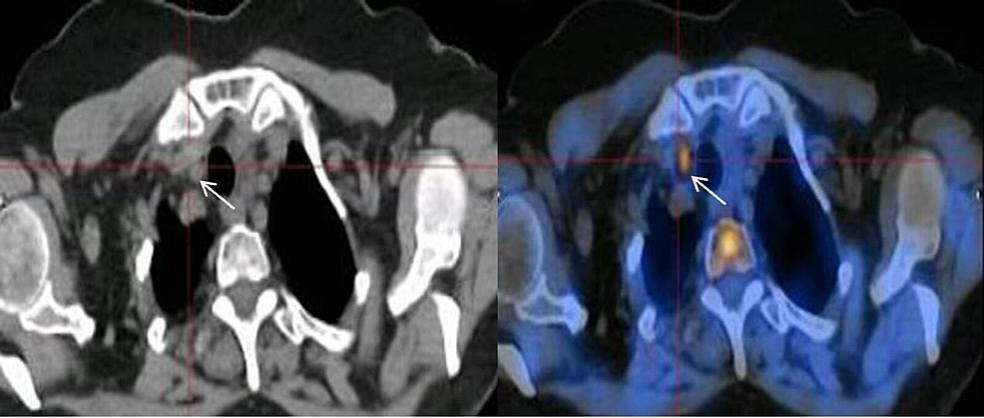
Fluorine-18-Fluorocholine positron emission tomography/computed tomography White arrows – prominent avidity in the posteroinferior region of the right thyroid lobe.

According to the ^99m^Tc-sestamibi scintigraphies results, the patient underwent excision of the left submandibular gland where the uptake focus was visible. No biochemical remission was achieved (albumin-corrected serum calcium 10.3 [8.8-10.2] mg/dL; serum PTH 147 [15.0-65.0] pg/mL) and the histopathological analysis of the surgical specimen showed the submandibular gland with no representation of a parathyroid gland. Once an abnormal parathyroid gland was not identified in cervical ultrasound and in scintigraphies, a ^18^F-Fluorocholine PET/CT was performed, which evidenced a small cervical nodule with prominent avidity for fluorocholine, compatible with a right parathyroid adenoma (Figure 2).

Intraoperative PTH reduction (121 pg/mL; 50 pg/mL; 28 pg/mL [0, 5, 15 minutes, respectively]) was accomplished after removal of the lesion. The histopathological analysis confirmed the excision of a parathyroid adenoma. The patient is currently cured based on biochemical evidence (Table 3) and no episodes of nephrolithiasis occurred after the surgery.

**Table 3:** Current laboratory evaluation

Parameters	Patient´s value	Reference range
Albumin-corrected serum calcium (mg/dL)	9.5	8.8-10.2
Phosphate (mg/dL)	2.8	2.7-4.5
Parathyroid hormone (pg/mL)	44.0	15.0-65.0
24-h urinary calcium (mg/24h)	284	100-320

## Discussion

The detection of the lesion by the ^18^F-Fluorocholine PET/CT led to an effective and safe (no surgery-related complications) parathyroidectomy, which that would not be possible with conventional morphofunctional assessment. The cost-effectiveness analysis favored the execution of such a costly diagnostic method, the ^18^F-Fluorocholine PET/CT, as a timely location of the abnormal parathyroid was vital to avoid a delayed treatment, in this patient with serious complications (nephrolithiasis and osteoporosis) related to the condition, which would be aggravated and recurred if HPT had been left untreated. In fact, an effective surgery permits the resolution of primary HPT and the avoidance of a panoply of hypercalcemia- and PTH overproduction-related symptoms (gastrointestinal and neuropsychiatric symptoms) and complications (decreased bone mineral density and fracture risk, and nephrolithiasis recurrence), respectively [5].

Choline-based PET tracers have been used with success in the investigation of well-differentiated prostate cancer [6]. Hyperfunctioning parathyroid glands were incidentally identified for the first time through ^11^C-choline and ^18^F-Fluorocholine PET/CT tracers in 2012 and 2013, respectively [7,8]. Since then, investigation emerged towards the accuracy of these techniques for localization of parathyroid adenoma and if they may eventually replace conventional ^99m^Tc-sestamibi scintigraphy.

In the last three years, a total of five systematic reviews and meta-analyses were published in this regard [9-13]. In all, ^18^F-Fluorocholine PET/CT showed a high preoperative detection rate of abnormal parathyroids. The most recent systematic review was published in 2020 and included 23 articles regarding a total of 1112 patients who performed ^18^F-Fluorocholine PET/CT [13]. It concluded that in populations with negative or equivocal conventional imaging findings, ^18^F-Fluorocholine PET/CT was more accurate than cervical ultrasound and ^99m^Tc-sestamibi scintigraphy in locating abnormal parathyroids, in patients with primary and recurrent HPT, regardless of the severity of the condition and the acquisition protocol used.

Recent technical developments provided high-resolution CT or magnetic resonance imaging that can detect smaller lesions. Four-dimensional contrast-enhanced CT merges standard multiplanar CT scanning with a fourth dimension consisting of changes in contrast attenuation over time, which offers comprehensive anatomical and functional data about altered parathyroids with a sensitivity of around 85%. However, high-level radiation exposure limits its usage in daily practice [14].

## Conclusions

^18^F-Fluorocholine PET/CT has revealed a promising role in primary HPT, being more accurate than conventional imaging methods in identifying abnormal parathyroids, especially smaller lesions. Thus, ^18^F-Fluorocholine PET/CT is an appropriate approach in selected cases, when conventional imaging techniques present equivocal findings. However, more powerful studies are needed to define it as the first-line diagnostic approach.
